# Multilocus genetics to reconstruct aeromonad evolution

**DOI:** 10.1186/1471-2180-12-62

**Published:** 2012-04-30

**Authors:** Frédéric Roger, Hélène Marchandin, Estelle Jumas-Bilak, Angeli Kodjo, the colBVH study group, Brigitte Lamy

**Affiliations:** 1 Laboratoire de Bactériologie-Virologie (UMR 5119 - Equipe Pathogènes et Environnements), Université Montpellier 1, 15, Avenue Charles Flahault, BP 14491, 34093, Montpellier Cedex 5, France; 2 Laboratoire de Bactériologie, Centre Hospitalier Régional Universitaire de Montpellier (Hôpital Arnaud de Villeneuve), 371, Avenue du Doyen Gaston Giraud, 34295, Montpellier Cedex 5, France; 3 Laboratoire d’Hygiène hospitalière, Centre Hospitalier Universitaire de Montpellier, 778, Rue de la Croix Verte, 34000, Montpellier, France; 4 CNRS UMR 5557 Ecologie microbienne, VetAgro Sup Campus vétérinaire de Lyon, 69280, Marcy-l’Étoile, France; 5 Groupe d’ Etude Français des Aeromonas (GFA), Lyon, France; 6 ColBVH, Collège de bactériologie, virologie et hygiène des hôpitaux généraux, Lyon, France

## Abstract

**Background:**

*Aeromonas* spp. are versatile bacteria that exhibit a wide variety of lifestyles. In an attempt to improve the understanding of human aeromonosis, we investigated whether clinical isolates displayed specific characteristics in terms of genetic diversity, population structure and mode of evolution among *Aeromonas* spp. A collection of 195 *Aeromonas* isolates from human, animal and environmental sources was therefore genotyped using multilocus sequence analysis (MLSA) based on the *dnaK*, *gltA*, *gyrB*, *radA*, *rpoB*, *tsf* and *zipA* genes.

**Results:**

The MLSA showed a high level of genetic diversity among the population, and multilocus-based phylogenetic analysis (MLPA) revealed 3 major clades: the *A. veronii*, *A. hydrophila* and *A. caviae* clades*,* among the eleven clades detected. Lower genetic diversity was observed within the *A. caviae* clade as well as among clinical isolates compared to environmental isolates. Clonal complexes, each of which included a limited number of strains, mainly corresponded to host-associated subsclusters of strains, i.e., a fish-associated subset within *A. salmonicida* and 11 human-associated subsets, 9 of which included only disease-associated strains. The population structure was shown to be clonal, with modes of evolution that involved mutations in general and recombination events locally. Recombination was detected in 5 genes in the MLSA scheme and concerned approximately 50% of the STs. Therefore, these recombination events could explain the observed phylogenetic incongruities and low robustness. However, the MLPA globally confirmed the current systematics of the genus *Aeromonas.*

**Conclusions:**

Evolution in the genus *Aeromonas* has resulted in exceptionally high genetic diversity. Emerging from this diversity, subsets of strains appeared to be host adapted and/or “disease specialized” while the *A. caviae* clade displayed an atypical tempo of evolution among aeromonads. Considering that *A. salmonicida* has been described as a genetically uniform pathogen that has adapted to fish through evolution from a variable ancestral population, we hypothesize that the population structure of aeromonads described herein suggested an ongoing process of adaptation to specialized niches associated with different degrees of advancement according to clades and clusters.

## Background

Aeromonads are ubiquitous free-living organisms found in aquatic environments with a strong ability to quickly colonize an exceptionally wide variety of habitats and hosts, ranging from hostile environments, such as polluted or chlorinated water, to leeches, insects, fish, mollusks, and mammals, including man [1]. They are opportunistic pathogens involved in various types of infections in a wide range of hosts. This versatility is supported by a large variety of genes involved in metabolic fitness and virulence; thus, *Aeromonas hydrophila* is referred to as a “jack-of-all-trades” [2]. Despite the adaptability of *A. hydrophila*, very few mobile genetic elements, which are usually associated with rapid adaptation, have been found in the complete genomic sequence of the pathogenic strain *A. hydrophila* ATCC 7966^T^[2].

Additionally, because some hosts may only be either colonized or infected, the concept that only specific subsets of *Aeromonas* strains within species might actually be pathogenic for humans was proposed [3,4]. In this setting, the question has arisen of whether isolates causing infectious diseases are exceptional and can be distinguished from other strains. Comparative analyses including environmental and clinical isolates showed that clinical strains are well differentiated from strains collected in the environment based on multilocus enzyme electrophoresis (MLEE) [5]. Other studies employing phenotypic, genotypic and virulence analyses have failed to distinguish isolates involved in infectious diseases from those that are not [3,6-8]. However, this situation is complex because the pathogenesis of *Aeromonas* infection is multifactorial and is associated with multiple sources of variability (e.g., a wide variety of virulence factor genes and the influence of environmental conditions); these studies are usually limited either by the sampling strategy applied (e.g., including a low number of isolates, species or types of infection), incomplete virulence factor analyses, or an absence of virulence gene expression analysis.

Overall, in the case of generalist opportunistic pathogens, which do not meet all of the criteria Koch’s postulate, the link between virulence-related genes and infection is not clearly established, and this opportunistic pathogenic behavior may instead be considered to represent an adaptation to human ecology [9-11]. There is evidence that genetic clusters can correspond to ecologically distinct populations and/or host-adapted populations, even when genes that are not related to virulence are considered [9,11-14].

In this context, in an attempt to improve the understanding of human aeromonosis, we investigated whether clinical isolates displayed specific characteristics among a large population of *Aeromonas* spp. from various origins. Because the 3 main *Aeromonas* species recovered from human clinical infectious diseases are *A. caviaeA. hydrophila* and *A. veronii* biovar *sobria*, we particularly focused on isolates belonging to these 3 taxa. The aim of this work was to determine the genetic characteristics, population structure and mode of evolution in a large population of aeromonads using a comparative approach that examined human, non-human animal and environmental strains. For this purpose, we developed a multilocus sequence analysis (MLSA) scheme specific for aeromonads, representing the third MLSA scheme to be described for this genus [15,16]. This strategy provided 4 new genes and produced new information on the mode of evolution, recombination rates and horizontal gene transfer in these species. This study, which was based on a large human clinical strain collection, provides interesting insight regarding the mode of evolution of aeromonads linked with human infection.

## Methods

### Bacterial strains

A total of 195 strains of *Aeromonas* spp., including 62 type and reference strains, were analyzed. The distribution of the origin of these strains was as follows: 115 human clinical strains, 39 non-human animal strains and 41 environmental strains (Table 1). Of the 115 human clinical isolates, 67 and 7 isolates were sampled in 2006 during a prospective study on aeromonosis involving 70 hospitals from mainland France and overseas territories, respectively [17]; 3 and 6 additional strains were isolated prior to 2006 from mainland France and overseas territories, respectively; 19 isolates were recovered from patients admitted to the Montpellier University Hospital between 2008 and 2010; and 5 strains were isolated in the USA, 2 in India, 2 in Taiwan, 1 in Bangladesh, 1 in New Zealand, 1 in Switzerland and 1 in the Czech republic. Clinical data were collected with the aim of specifying the clinical implications of the strains, i.e., whether they were involved in an infectious process or in host colonization. Of the 80 non-human isolates, 33 were isolated in France from a wastewater treatment lagoon (n = 11), recreational lake water (n = 13), healthy snails (n = 7) or fish (n = 2), and 36 strains were isolated from diverse sources among 13 countries worldwide, mainly in Europe and North America, while the countries of origin of 11 strains were unknown. The strain collection included type strains of 28 species and a representative strain of hybridization group (HG) 11 (Table 1).

**Table 1:** Typing data and origin of *Aeromonas* strains analyzed in the study

**MLPA clade or isolated taxon**	**Strain**	**ST**	**CC (5)^a^**	**CC (4)^b^**	**Allelic profile**							**Origin**	**Infection (I) or colonization (C)**	**Region, country and year of isolation**
					***dnaK***	***gltA***	***gyrB***	***radA***	***rpoB***	***tsf***	***zipA***			
*A. hydrophila* (n=35)	BVH3	2	-	A	2	2	2	2	2	2	2	Human, Wound	I	Cahors, Fr, 2006
	BVH14	7	-	A	7	2	7	7	2	2	2	Human, Wound	I	Le Mans, Fr, 2006
	*A. hydrophila* subsp. *hydrophila* CCM 2278	7	-	A	7	2	7	7	2	2	2	Non-human, Red-legged Frog	I	California, USA, 1963
	BVH29	18	-	-	18	15	17	17	13	15	17	Human, Wound	I	Reunion Island, Fr, 2006
	BVH30	18	-	-	18	15	17	17	13	15	17	Human, Stool	I	Langres, Fr, 2006
	BVH35	23	-	-	23	19	21	22	18	12	20	Human, Wound	C	La Roche sur Yon, Fr, 2006
	BVH99	23	-	-	23	19	21	22	18	12	20	Human, NA	ND	Brest, Fr, ND
	BVH25a	15	-	-	15	13	14	14	10	13	14	Human, Respiratory tract	I	Saint-Brieux, Fr,2006
	BVH27b	15	-	-	15	13	14	14	10	13	14	Human, Wound	I	Reunion Island, Fr, 2006
	AK204	15	-	-	15	13	14	14	10	13	14	Non-human, Snail	I	Angers, Fr, 1995
	BVH2	1	-	-	1	1	1	1	1	1	1	Human, Wound	I	Cahors, Fr, 2006
	BVH12	5	-	-	5	5	5	5	5	5	5	Human, Eyes infection	I	Cherbourg, Fr, 2006
	BVH24	14	-	-	14	12	13	13	9	12	13	Human, Wound	I	Saint-Brieux, Fr,2006
	BVH33	21	-	-	21	18	20	20	16	2	20	Human, Wound	I	La Rochelle, Fr, 2006
	BVH34	22	-	-	22	15	17	21	17	18	21	Human, Urine	C	La Roche sur Yon, Fr, 2006
	BVH36	24	-	-	24	20	22	23	19	5	22	Human, Wound	I	La Roche sur Yon, Fr, 2006
	BVH41	28	-	-	28	24	26	27	23	22	26	Human, Wound	I	Vannes, Fr, 2006
	BVH42	29	-	-	29	25	27	28	24	12	27	Human, Wound	I	Périgueux, Fr, 2006
	BVH45	32	-	-	32	28	30	14	10	25	30	Human, Wound	I	Périgueux, Fr, 2006
	BVH64	48	-	-	47	15	44	42	35	35	42	Human, Wound	I	Belfort, Fr, 2006
	BVH72	55	-	-	52	42	49	48	39	12	46	Human, Blood	ND	Martinique Island, Fr, ND
	BVH75	57	-	-	53	44	51	49	2	12	48	Human, Respiratory tract	I	Saint-Etienne, Fr,2006
	BVH93	68	-	-	63	52	60	57	47	45	14	Human, Wound	I	Cahors, Fr, 2006
	BVH96	70	-	-	65	54	62	59	48	47	56	Human, Wound	C	Bourg en Bresse, Fr, 2006
	BVH97	71	-	-	66	55	63	1	49	48	57	Human, Wound	I	Bourg en Bresse, Fr, 2006
	ADV105	76	-	-	69	58	67	63	51	49	60	Human, Stool	ND	Montpellier, Fr, 2008
	AK203	93	-	-	86	70	81	76	60	61	14	Non-human, Snail	I	Angers, Fr, 1995
	AK218	95	-	-	88	72	83	78	47	49	74	Environment, Waste water treatment lagoon	-	Montracol, Fr, 2006
	AK235	105	-	-	97	79	93	88	66	67	81	Environment, Waste water treatment lagoon	-	Montracol, Fr, 2006
	*A. hydrophila* subsp. *hydrophila* CECT 839^T^	130	-	-	120	102	114	108	79	81	22	Environment, Tin of milk with a fishy odor	-	NA, NA, NA
	*A. hydrophila* subsp. *ranae* CIP 107985	131	-	-	121	103	115	109	80	82	101	Non-human, Frog	I	NA, Thaïland, NA
	*A. hydrophila* CECT 5734	163	-	-	150	132	144	137	104	12	127	Non-human, Fish	I	Valencia, Spain, 1987
	*A. hydrophila* subsp. *hydrophila* CCM 2280	171	-	-	69	139	152	145	111	115	134	Non-human, Snake	-	NA, NA, 1963
	*A. hydrophila* subsp. *hydrophila* CCM 2282	172	-	-	158	140	153	146	47	116	135	Non-human, Nile Monitor	ND	NA, NA, 1963
	*A. hydrophila* subsp. *hydrophila* CCM 4528	174	-	-	160	15	17	148	13	118	137	Human, Stool	ND	NA, Czech Republic, 1993
*A. veronii* (n=71)	BVH22	13	-	-	13	11	12	4	8	11	12	Human, Wound	I	Alès, Fr, 2006
	BVH23	13	-	-	13	11	12	4	8	11	12	Human, Wound	I	Saint-Brieux, Fr,2006
	BVH25b	13	-	-	13	11	12	4	8	11	12	Human, Respiratory tract	I	Saint-Brieux, Fr,2006
	BVH26a	13	-	-	13	11	12	4	8	11	12	Human, Wound	I	Saint-Brieux, Fr,2006
	BVH27a	13	-	-	13	11	12	4	8	11	12	Human, Wound	I	Reunion Island, Fr,2006
	BVH28a	13	-	-	13	11	12	4	8	11	12	Human, Wound	I	Reunion Island, Fr,2006
	BVH61	46	5	D	46	29	31	31	34	34	40	Human, Stool	I	Antibes, Fr,2006
	BVH71	54	5	D	46	29	31	31	26	34	40	Human, Stool	ND	Martinique Island, Fr, ND
	BVH47	33	-	D	33	29	31	31	26	16	31	Human, Blood	I	Roubaix, Fr,2006
	ADV102	33	-	D	33	29	31	31	26	16	31	Human, Stool	ND	Montpellier, Fr, 2008
	BVH18	10	-	-	10	9	10	10	7	9	9	Human, Wound	I	Villeneuve sur Lot, Fr, 2006
	AK249	10	-	-	10	9	10	10	7	9	9	Environment, Water lake		Annecy, Fr, 1998
	ADV129	85	8	H	78	64	74	69	56	56	67	Human, Stool	ND	Montpellier, Fr, 2009
	ADV133	89	8	H	82	64	74	69	56	56	67	Human, Wound	I	Montpellier, Fr, 2010
	BVH 90	66	7	G	61	6	58	55	45	43	53	Human, Stool	I	Dunkerque, Fr, 2006
	AK236	106	7	G	61	6	58	55	45	68	53	Environment, Water lake	-	Annecy, Fr, 1998
	BVH37	25	-	-	25	21	23	24	20	19	23	Human, Blood	I	La Roche sur Yon, Fr, 2006
	BVH46	25	-	-	25	21	23	24	20	19	23	Human, Blood	I	Roubaix, Fr, 2006
	BVH56	42	4	E	42	36	40	24	32	6	23	Human, Blood	I	Versailles, Fr, 2006
	ADV101	74	4	E	42	57	40	24	32	19	23	Human, Stool	ND	Montpellier, Fr, 2008
	*A. veronii* bv. *veronii* CECT 4257^T^	143	-	-	131	114	125	120	11	19	110	Human, Respiratory tract	I	Michigan, USA, NA
	*A. veronii* CCM 4360	143	-	-	131	114	125	120	11	19	110	Human, Stool	I	Connecticut, USA, 1984
	BVH6	4	-	-	4	4	4	4	4	4	4	Human, Wound	I	Cahors, Fr, 2006
	BVH13	6	-	-	6	6	6	6	4	6	6	Human, Blood	I	Le Mans, Fr, 2006
	BVH26b	16	-	-	16	11	15	15	11	6	15	Human, Wound	I	Saint-Brieux, Fr,2006
	BVH31	19	-	-	19	16	18	18	14	16	18	Human, Bile	I	La Rochelle, Fr, 2006
	BVH32	20	-	-	20	17	19	19	15	17	19	Human, Stool	C	La Rochelle, Fr, 2006
	BVH44	31	-	-	31	27	29	30	20	24	29	Human, Wound	I	Périgueux, Fr, 2006
	BVH49	35	-	-	35	30	33	33	20	27	33	Human, Stool	I	Chalon sur Saone, Fr, 2006
	BVH50	36	-	-	36	31	34	34	28	6	34	Human, Respiratory tract	I	Chalon sur Saone, Fr, 2006
	**BVH53**	39	-	-	39	34	**37**	37	31	30	29	Human, Blood	I	Saint Denis, Fr, 2006
	BVH54	40	-	-	40	35	38	38	4	31	37	Human, Respiratory tract	I	Saint Denis, Fr, 2006
	BVH59	44	-	-	44	37	41	39	33	19	38	Human, Blood	I	Le Havre, Fr, 2006
	BVH60	45	-	-	45	38	42	40	20	33	39	Human, Stool	I	Antibes, Fr, 2006
	BVH73	56	-	-	39	43	50	37	40	38	47	Human, Blood	ND	Martinique Island, Fr, NA
	BVH77	58	-	-	54	45	52	50	41	19	49	Human, Stool	C	Aix en Provence, Fr, 2006
	BVH79	59	-	-	55	46	53	51	4	19	37	Human, Wound	I	Aix en Provence, Fr, 2006
	BVH80	60	-	-	56	47	54	52	42	39	50	Human, Stool	I	Aix en Provence, Fr, 2006
	BVH95	69	-	-	64	53	61	58	20	46	55	Human, Wound	I	Bourg en Bresse, Fr, 2006
	ADV103	75	-	-	68	29	66	62	26	34	59	Human, Stool	ND	Montpellier, Fr, 2008
	ADV109	78	-	-	71	60	69	65	53	51	61	Human, Stool	ND	Montpellier, Fr, 2008
	ADV119	80	-	-	73	61	70	66	53	52	62	Human, Stool	ND	Montpellier, Fr, 2009
	ADV125	83	-	-	76	63	72	68	54	54	65	Human, Stool	ND	Montpellier, Fr, 2009
	ADV127	84	-	-	77	11	73	68	55	55	66	Human, Stool	ND	Montpellier, Fr, 2009
	ADV130	86	-	-	79	11	75	70	33	17	68	Human, Blood	ND	Montpellier, Fr, 2010
	ADV131	87	-	-	80	65	76	71	11	19	69	Human, Respiratory tract	ND	Montpellier, Fr, 2009
	ADV135	90	-	-	83	67	78	73	57	58	23	Human, Stool	ND	Montpellier, Fr, 2010
	ADV137b	91	-	-	84	68	79	74	58	59	71	Human, Respiratory tract	ND	Montpellier, Fr, 2010
	AK219	96	-	-	89	27	84	79	11	62	75	Environment, Waste water treatment lagoon	-	Montracol, Fr, 2006
	AK222	97	-	-	90	73	85	80	62	19	76	Environment, Waste water treatment lagoon	-	Montracol, Fr, 2006
	AK226	99	-	-	92	75	87	82	8	63	12	Environment, Waste water treatment lagoon	-	Montracol, Fr, 2006
	AK227	100	-	-	93	76	88	83	34	64	29	Environment, Waste water treatment lagoon	-	Montracol, Fr, 2006
	AK232	103	-	-	95	11	91	86	64	65	79	Environment, Waste water treatment lagoon	-	Montracol, Fr, 2006
	AK237	107	-	-	98	80	94	51	67	19	82	Environment, Water lake	-	Annecy, Fr, 1998
	AK238	108	-	-	99	81	95	89	34	69	83	Environment, Water lake	-	Annecy, Fr, 1998
	AK239	109	-	-	100	82	73	68	55	6	84	Environment, Water lake	-	Annecy, Fr, 1998
	AK240	110	-	-	101	83	96	90	20	19	85	Environment, Water lake	-	Annecy, Fr, 1998
	AK241	111	-	-	102	84	97	91	20	56	86	Non-human, Snail	I	Angers, Fr, 1995
	AK242	112	-	-	25	85	98	92	68	19	23	Environment, Water lake	-	Annecy, Fr, 1998
	AK243	113	-	-	103	86	98	93	20	70	87	Non-human, Snail	I	Angers, Fr, 1995
	AK244	114	-	-	104	87	99	94	69	19	23	Environment, Water lake	-	Annecy, Fr, 1998
	AK246	116	-	-	106	89	101	95	71	19	12	Non-human, Snail	I	Angers, Fr, 1995
	AK247	117	-	-	107	90	102	96	58	72	89	Environment, Water lake	-	Annecy, Fr, 1998
	AK248	118	-	-	108	91	103	97	34	73	90	Environment, Water lake	-	Annecy, Fr, 1998
	**AK250**	119	-	-	109	27	**83**	98	11	19	75	Environment, Water lake	-	Annecy, Fr, 1998
	AK251	120	-	-	110	92	104	99	20	19	91	Environment, Water lake	-	Annecy, Fr, 1998
	***A. culicicola*CIP 107763**^**T**^	124	-	-	114	96	108	4	33	**77**	95	Non-human, Mosquito midgut	ND	Pune, India, 1997
	*A. ichthiosmia* CECT 4486^T^	132	-	-	122	104	116	110	81	19	102	Environment, Surface water	-	NA, Germany, 1986
	*A. veronii* bv. *sobria* LMG 13067	144	-	-	132	115	126	121	8	93	12	Non-human, Frog	I	Connecticut, USA, NA
	*A. veronii* CECT 4902	161	-	-	148	131	143	136	103	108	126	Environment, NA	-	NA, Germany, 1993
	*A. veronii* CECT 7059	164	-	-	151	133	145	138	33	109	39	Environment, Drinking water	-	Zaragoza, Spain, 2002
*A. caviae* (n=34)	BVH16	9	1	B	9	8	9	9	3	8	8	Human, Respiratory tract	C	Rambouillet, Fr, 2006
	BVH57	43	1	B	43	8	9	9	3	32	8	Human, Blood	I	Versailles, Fr, 2006
	BVH63	47	6	F	12	10	43	41	3	10	41	Human, Blood	I	Macon, Fr, 2006
	BVH84	47	6	F	12	10	43	41	3	10	41	Human, Stool	I	Aix en Provence, Fr, 2006
	BVH98	72	-	F	12	10	64	60	37	10	41	Human, Wound	I	Brest, Fr, NA
	ADV118	79	6	F	72	10	43	8	3	10	41	Human, Wound	I	Montpellier, Fr, 2009
	ADV121	81	-	F	74	10	43	8	3	3	63	Human, Stool	ND	Montpellier, Fr, 2009
	BVH48	34	2	C	34	10	32	32	27	26	32	Human, Vagina	C	Monceau les mines, Fr, 2006
	*A. caviae* CCUG 48892	175	2	C	34	10	32	32	27	3	32	Environment, Water		Uppsala, Sweden, 2004
	BVH19	11	-	C	11	10	3	11	3	10	10	Human, Vagina	C	Villeneuve sur Lot, Fr, 2006
	BVH81	61	-	C	34	10	3	11	3	26	32	Human, Stool	C	Aix en Provence, Fr, 2006
	BVH66	50	-	C	34	10	46	44	37	26	32	Human, Wound	I	Martinique Island, Fr, 2006
	BVH55	41	3	C	41	10	39	12	3	26	32	Human, Stool	I	Saint-Denis, Fr, 2006
	BVH87	64	3	C	59	10	39	12	3	26	32	Human, Stool	I	Aix en Provence, Fr, 2006
	BVH4	3	-	-	3	3	3	3	3	3	3	Human, Wound	I	Cahors, Fr, 2006
	BVH15	8	-	-	8	7	8	8	6	7	7	Human, Blood	I	Grasse, Fr, 2006
	BVH20	12	-	-	12	10	11	12	3	8	11	Human, Stool	I	Gonesse, Fr, 2006
	BVH51	37	-	-	37	32	35	35	29	28	35	Human, Blood	I	Monaco, Fr, 2006
	BVH52	38	-	-	38	33	36	36	30	29	36	Human, Blood	I	Monaco, Fr, 2006
	BVH67	51	-	-	49	32	47	45	3	8	35	Human, Stool	ND	Martinique Island, Fr, NA
	BVH85	62	-	-	57	48	55	11	3	40	8	Human, Stool	I	Aix en Provence, Fr, 2006
	BVH86	63	-	-	58	49	56	53	43	41	51	Human, Stool	C	Aix en Provence, Fr, 2006
	BVH100	73	-	-	67	56	65	61	50	26	58	Human, Wound	ND	Brest, Fr, ND
	ADV106	77	-	-	70	59	68	64	52	50	35	Human, Stool	ND	Montpellier, Fr, 2008
	ADV124	82	-	-	75	62	71	67	3	53	64	Human, Stool	ND	Montpellier, Fr, 2009
	AK223	98	-	-	91	74	86	81	3	8	77	Environment, Waste water treatment lagoon	-	Montracol, Fr, 2006
	AK229	101	-	-	34	77	89	84	37	3	78	Environment, Waste water treatment lagoon	-	Montracol, Fr, 2006
	AK231	102	-	-	94	78	90	85	63	26	32	Environment, Waste water treatment lagoon	-	Montracol, Fr, 2006
	AK234	104	-	-	96	10	92	87	65	66	80	Environment, Waste water treatment lagoon	-	Montracol, Fr, 2006
	AK245	115	-	-	105	88	100	11	70	71	88	Environment, Water lake	-	Annecy, Fr, 1998
	***A. caviae*CECT 838**^**T**^	123	-	-	113	95	107	102	74	76	**94**	Non-human, Guinea pig	I	NA, USA, NA
	*A. hydrophila* subsp. *anaerogenes* CECT 4221	128	-	-	118	100	112	106	50	3	99	Environment, Used oil emulsion	-	NA, USA, NA
	*A. caviae* CECT 4222	154	-	-	142	78	136	131	37	103	35	Environment, Sewage	-	NA, NA, 1954
	*A. caviae* CECT 4226	155	-	-	118	125	137	11	50	3	120	Environment, Used oil emulsion	-	NA, USA, 1953
*A. piscicola* (n=3)	*A. piscicola* LMG 24783^T^	151			139	122	133	128	95	100	117	Non-human, Salmon	I	Gallicia, Spain, 2005
	*A. sobria* CECT 4333	156	9	J	143	126	138	132	98	104	121	Non-human, Diseased elver	I	Valencia, Spain, NA
	*Aeromonas* sp. CECT 5177	162	9	J	149	126	138	132	98	104	121	Environment, Drinking water	-	Eeklo, Belgium, 1996
*A. salmonicida* (n=8)	*A. salmonicida* subsp. *achromogenes* CIP 104001	136	-	I	126	107	120	114	85	86	94	Non human, Trout	ND	Aberdeen, UK, 1963
	*A. salmonicida* subsp. *masoucida* CIP 103210	137	-	I	126	108	120	115	85	87	105	Non human, Fish blood	I	NA, NA, 1969
	*A. salmonicida* subsp. *smithia* CIP 104757	137	-	I	126	108	120	115	85	87	105	Non human, Fish ulcer	I	NA, UK, NA
	*A. salmonicida* subsp. *salmonicida* CIP 103209^T^	139	-	I	126	110	120	114	85	89	105	Non human, Diseased salmon	I	Cletter river, UK, 1953
	**BVH39**	26	-	-	**26**	22	24	25	21	20	24	Human, Wound	C	Vannes, Fr, 2006
	*A. salmonicida* subsp. *pectinolytica* CIP 107036	138	-	-	127	109	121	116	86	88	106	Environment, River water		Buenos Aires, Argentina, NA
	*A. salmonicida* CCM 1150	168	-	-	155	136	149	142	108	112	131	Non human, Fish	ND	NA, Czech Republic, 1961
	*A. salmonicida* CCM 1275	170	-	-	157	138	151	144	110	114	133	Fish	ND	NA, Czech Republic, 1961
*A. allosaccharophila* (n=3)	BVH88	65	-	-	60	50	57	54	44	42	52	Human, Blood	I	Dunkerque, Fr, 2006
	*A. allosaccharophila* CECT 4199^T^	121	-	-	111	93	105	100	72	74	92	Non-human, Fish	I	Valencia, Spain, 1991
	*A. sobria* CECT 4053	153	-	-	141	124	135	130	97	102	119	Environment, Activated sludge		Stockholm, Sweden, 1978
*A. sobria* (n=5)	*A. sobria* CECT 4245^T^	141	-	-	129	112	123	118	88	91	108	Non-human, Fish	ND	NA, Fr, 1974
	*Aeromonas* sp. CECT 4816	157	-	-	144	127	139	133	99	105	122	Non-human, Fish	ND	NA, NA, 1993
	*Aeromonas* sp. CECT 4817	158	-	-	145	128	140	134	100	106	123	Non-human, Fish	ND	NA, NA, 1993
	*Aeromonas* sp. CECT 4818	159	-	-	146	129	141	135	101	107	124	Non-human, Fish	ND	NA, NA, 1993
	*A. sobria* CECT 4821	160	-	-	147	130	142	118	102	91	125	Non-human, Fish	ND	NA, NA, 1993
*A. aquariorum* (n=8)	BVH28b	17	-	-	17	14	16	16	12	14	16	Human, Wound	I	Reunion Island, Fr, 2006
	BVH43	30	-	-	30	26	28	29	25	23	28	Human, Wound	I	Périgueux, Fr, 2006
	BVH65	49	-	-	48	39	45	43	36	36	43	Human, Blood	I	Martinique Island, Fr, 2006
	BVH68	52	-	-	50	40	48	46	12	23	44	Human, NA	ND	Martinique Island, Fr, ND
	BVH70	53	-	-	51	41	28	47	38	37	45	Human, NA	ND	Martinique Island, Fr, ND
	ADV132	88	-	-	81	66	77	72	25	57	70	Human, Wound	I	Montpellier, Fr, 2010
	*A. hydrophila* subsp. *dhakensis* CIP 107500	129	-	-	119	101	113	107	78	23	100	Human, Stool	I	NA, Bangladesh, NA
	*A. aquariorum* CECT 7289^T^	145	-	-	133	116	127	122	89	94	111	Non-human, Fish	ND	NA, Portugal, 2003
*A. media* (n=6)	BVH40	27	-	-	27	23	25	26	22	21	25	Human, Stool	C	Vannes, Fr, 2006
	AK202	92	-	-	85	69	80	75	59	60	72	Non-human, Snail	I	Angers, Fr, 1995
	AK211	94	-	-	87	71	82	77	61	60	73	Non-human, Snail	I	Angers, Fr, 1995
	***A. media*CECT 4232**^**T**^	134	-	-	124	71	118	112	83	84	**97**	Environment, Fish farm effluent water	-	NA, UK, NA
	*Aeromonas* sp. CECT 7111	167	-	-	154	71	148	141	107	60	130	Non-human, Oyster	-	Barcelona, Spain, NA
	***A. media*CCM 4242**	173	-	-	159	141	154	147	59	117	**136**	Environment, River water	-	NA, Czech Republic, 1991
*A. tecta* (n=3)	*A. tecta* CECT 7082^T^	146	-	-	134	117	128	123	90	95	112	Human, Stool	ND	Ticino, Switzerland, NA
	*Aeromonas* sp. CECT 7081	165	-	-	152	134	146	139	105	110	128	Non-human, Fish	ND	Ticino, Switzerland, 1983
	*Aeromonas* sp. CECT 7083	166	-	-	153	135	147	140	106	111	129	Environment, Tap water	-	Ticino, Switzerland, 1993
*A. jandaei*	BVH92	67	-	-	62	51	59	56	46	44	54	Human, Urine	I	Toulouse, Fr, 2006
(n=2)	*A. jandaei* CECT 4228^T^	133	-	-	123	105	117	111	82	83	103	Human, Stool	ND	Oregon, USA, 1980
*A. enteropelogenes*	***A. enteropelogenes*CECT 4487**^**T**^	126	-	-	116	98	**110**	104	76	79	97	Human, Stool	ND	NA, India, NA
*A. trota*	***A. trota*CECT 4255**^**T**^	142	-	-	130	113	**124**	119	76	92	109	Human, Stool	ND	Varasani, India, NA
*A. bestiarum*	*A. bestiarum* CECT 4227^T^	122	-	-	112	94	106	101	73	75	93	Non-human, Fish	ND	NA, Fr, 1974
*A. encheleia*	*A. encheleia* CECT 4342^T^	125	-	-	115	97	109	103	75	78	96	Non-human, Fish	I	Valencia, Spain, 1987
HG11	HG11 CECT 4253	147	-	-	135	118	129	124	91	96	113	Human, Wound	I	New Zealand, 1983
*A. eucrenophila*	*A. eucrenophila* CECT 4224^T^	127	-	-	117	99	111	105	77	80	98	Non-human, Freshwater fish	ND	NA, NA, NA
*A. fluvialis*	***A. fluvialis*LMG 24681**^**T**^	149	-	-	137	**120**	131	126	93	**98**	115	Environmental, River water	-	Girona, Spain, NA
*A. popoffii*	*A. popoffi* CIP 105493^T^	135	-	-	125	106	119	113	84	85	104	Environmental, Water	-	Oelegem, Belgium, 1993
*A. sanarellii*	*A. sanarellii* LMG 24682^T^	152	-	-	140	123	134	129	96	101	118	Human, Wound	I	NA, Taïwan, 2000
*A. schubertii*	*A. schubertii* CECT 4240^T^	140	-	-	128	111	122	117	87	90	107	Human, Wound	I	Texas, USA, 1981
*A. diversa*	HG13 CECT 4254^T^	148	-	-	136	119	130	125	92	97	114	Human, Wound	I	Louisiana, USA, NA
*A. taiwanensis*	*A. taiwanensis* LMG 24683^T^	150	-	-	138	121	132	127	94	99	116	Human, Wound	I	NA, Taïwan, 2000
Unknown taxon	***A. bestiarum*CCM 1271**	169	-	-	156	137	150	143	109	**113**	132	Non-human, Gold fish	ND	NA, NA, NA
*A. bivalvium*	*A. bivalvium* CECT 7113^T^	-	-	-	161	142	155	-	112	119	138	Non-human, Cockles	-	Barcelona, Spain, 1997
*A. molluscorum*	*A. molluscorum* CIP 108876^T^	-	-	-	-	143	156	-	113	120	139	Non-human, Wedge-shells	-	Barcelona, Spain, 1997
*A. simiae*	*A. simiae* CIP 107798^T^	-	-	-	162	144	157	-	114	121	140	Non-human, Healthy monkey	-	NA, Mauritus, 1999
*A. rivuli*	*A. rivuli* DSM 22539^T^	-	-	-	-	145	158	149	115	122	141	Environment, Karst hardwater creek	-	Westerhöfer Bach, Germany, NA

For each strain, the table presents the results of the multilocus phylogenetic analysis (MLPA); the result of the multilocus sequence analysis (strain ST number and belonging to a clonal complex); the allelic number for each of the 7 genes; and the characteristics of strain isolation (origin, colonizing or infecting status, spatio-temporal origin).

^a^CC(5): clonal complex defined by at least 5 identical alleles.

^b^CC(4): clonal complex defined by at least 4 identical alleles.

ND: not determined; NA: not available; -: not applicable.

Names of strains and alleles concerned by recombination detected by phylogenetic incongruities are in bold type.

All bacteremia originated from the gut [17].

### Pulsed-field gel electrophoresis (PFGE)-restriction fragment length polymorphism (RFLP) analysis

Genomic DNA was prepared in agarose plugs as previously described [18] starting from a fresh culture on Trypticase Soja agar medium. After *Aeromonas* suspensions in 2 ml of Tris-NaCl buffer (1.0 M Tris base, 1.0 M NaCl, pH 7.6) were adjusted to an optical density of 1.5 at 650 nm, they were centrifuged (10,000 g for 1 min), 1 ml of the supernatant was then discarded, and the pellet was resuspended (final concentration 2:1). DNA was digested at 25°C with 40 U of *Swa*I (New England BioLabs, Hertfordshire, United Kingdom). The *Swa*I fragments were separated in a 1% agarose gel via PFGE using a CHEF-DRIII apparatus (Bio-Rad Laboratories, Hercules, CA) and 0.5X Tris-Borate-EDTA (TBE) buffer containing 50 μM thiourea at 5.5 V/cm and 10°C with pulse ramps of 100 to 5 s for 48 h. A lambda concatemer (Biolabs) was used as the size standard. The gel was stained with ethidium bromide and photographed under UV light. The PFGE profiles, known as pulsotypes, were compared visually by numbering both the shared and the distinct DNA fragments.

### Gene amplification and sequencing

The complete genomic sequences of *A. hydrophila* subsp. *hydrophila* ATCC 7966^T^ and *A. salmonicida* subsp. *salmonicida* A449 [GenBank accession numbers NC_008570 and NC_009348, respectively] were used employed as references for gene selection and primer design. The primers used in this study are described in Table 2. Genomic DNA was obtained using the Aquapure DNA extraction kit (EpiCentre, Madison, WI). PCR was carried out in a 50 μL reaction mixture containing 200 nM of each primer (Sigma Genosys), 200 μM of each deoxynucleoside triphosphate (dNTP) (Euromedex, Mundolsheim, France), 2 mM MgCl_2_, and 2.5 U of *Taq* DNA polymerase (Promega, Madison, WI) in the appropriate reaction buffer and 50 ng of genomic DNA as the template. The amplification conditions were as follows: initial denaturation for 4 min at 94°C, followed by 35 amplification cycles as indicated in Table 2 and a final extension step at 72°C for 10 min. *zipA* amplification required specific conditions for some *A. caviae* and *A. media* isolates included in this study, such as a 4 mM MgCl_2_ concentration and a primer hybridization temperature of 50°C (*A. caviae*).

**Table 2:** Primers and amplification conditions

**Locus**	**Function**	**Putative gene product**	**Gene size**^a^**(bp)**	**Locus position**^a^**(bp)**	**Primer**^b^	**Primer sequence 5’-3’**	**Sequence length amplified (alignment length) (bp)**	**Amplification conditions**
*dnaK*	Stress response	Heat shock 70 kDa protein (HSP 70)	1,928	3357741	DnaK-F DnaK-R	ATGAAGAAGACCGCCGAAG TGCAGCACGTGAATGGTC	816 (816)	45s at 94°C; 45s at 63°C; 45s at 72°C
*gltA*	Glycolytic pathway	Type II citrate synthase	1,286	2098162	GltA-F GltA-R	TTCCGTCTGCTCTCCAAGAT GCAGCGGATCCTTGATCT	462 (462)	45s at 94°C; 45s at 63°C; 45s at 72°C
*gyrB*	Replication	Subunit DNA gyrase	2,411	3883	GyrB3F GyrB14R	(Yáñez, 2003)	783-786 (792)	45s at 94°C; 45s at 65°C; 45s at 72°C
*radA*	DNA repair	DNA repair protein, RadA	1,364	4108941	RadA-F RadA-R	ATGCATCACCTGGATGGAGT TGCCTATGTTTGTACCGAATG	405-414 (420)	45s at 94°C; 45s at 60°C; 45s at 72°C
*rpoB*	Transcription	DNA-directed RNA polymerase β subunit	4,091	4466890	Pasrpob-L Rpob-R	(Korczak, 2004)	426 (426)	45s at 94°C; 45s at 65°C; 45s at 72°C
*tsf*	Protein translation	Elongation factor Ts	881	1272267	Tsf-F Tsf-R	CGCTGGCATGATGGATTG GATGCCTTCGCCCACTTC	702 (702)	45s at 94°C; 45s at 65°C; 45s at 72°C
*zipA*	Cell division	Cell division protein ZipA	1,112	1337458	ZipA-F ZipA-R	TTGCGTTACATCTTGGTTGC ATCCGGTACGGATTGAAGGT	429-507 (537)	1min at 94°C; 45s at 55°C^*c*^; 1min at 72°C

^a^Gene start codon position on the circular chromosome sequence of *A. hydrophila* subsp. *hydrophila* ATCC 7966^T^ (accession number NC_008570).

^b^F, forward primer; R, reverse primer.

^c^Some strains belonging to *A. caviae* and *A. media* clades were amplified using a 4 mM MgCl_2_ concentration and a primer hybridization temperature of 50°C (*A. caviae*).

The PCR products and a molecular weight marker (phage phiX DNA digested with *Hae*III, New England BioLabs) were separated in 1.5% agarose gels in 0.5X TBE buffer. The products were then sequenced using forward amplification primers (Table 2) in an ABI 3730XL automatic sequencer (Beckman Coulter Genomics, United Kingdom).

### Phylogenetic analysis

Gene sequences were codon aligned using the ClustalW application within the Bioedit Sequence Alignment Editor [19]. The sizes of the codon-aligned sequences that were used for further analyses are indicated in Table 2. Phylogenetic analyses were performed for each of the 7 gene sequences and for a manually concatenated sequence. Gaps in concatenated sequences were deleted with Bioedit. The sequences were converted in Phylip format using the ReadSeq online program (http://searchlauncher.bcm.tmc.edu/seq-util/readseq.html). A distance-based phylogenetic tree was reconstructed using the neighbor-joining method implemented in Neighbor from the PHYLIP package v3.66 [20]. Distances were calculated using FastDist. The K2P substitution model was selected for the analysis plus a gamma parameter fixed to 2. Distance bootstrap support was calculated after 1000 reiterations. The analysis was performed online on the site http://www.phylogeny.fr. For the maximum likelihood (ML) method-based phylogeny, evolutionary distance was analyzed with the PhyML v3.0 program [21] using GTR, with a gamma distribution parameter estimated from the dataset and invariant sites as a substitution model. This substitution model was determined to be the most appropriate by ModelTest [22]. ML bootstrap support was calculated after 100 reiterations.

### Multilocus sequence analysis

For each locus, each allele was assigned a distinct arbitrary number using a nonredundant database program available at http://www.pubmlst.org. The combination of allele numbers for each isolate defined the sequence type (ST).

Allele profiles were analyzed using eBURST v3 software [23] to determine the clonal complexes (CCs) defined as sets of related strains that share at least 5 identical alleles at the 7 loci. A complementary eBURST analysis was conducted to determine the CCs sharing at least 4 identical alleles at the 7 loci.

The program LIAN 3.5 [24], available at http://www.pubmlst.org, was used to calculate the standardized index of association (sIA) to test the null hypothesis of linkage disequilibrium, the mean genetic diversity (H) and the genetic diversity at each locus (h). The number of synonymous (dS) and non-synonymous (dN) substitutions per site was determined from codon-aligned sequences using Sequence Type Analysis and Recombinational Tests Version 2 (START2) software [25]. Other genetic analyses, including the determination of allele and allelic profile frequencies, mol% G + C content and polymorphic site numbering, were also carried out using START2 software. A distance matrix in nexus format was generated from the set of allelic profiles and then used for decomposition analyses with SplitsTree 4.0 software [26]. Recombination events were detected from the aligned ST concatenated sequences using the RDP v3.44 [27] software package with the following parameters: general (linear sequence, highest *P* value of 0.05, Bonferroni correction), RDP (no reference, window size of 8 polymorphic sites, 0-100% sequence identity range), GENECONV (scan triplets, G-scale of 1), Bootscan (window size of 200 bp, step size of 20 bp, 70% cutoff, F84 model, 100 bootstrap replicates, binomial *P* value), MAxChi (scan triplets, fraction of variable sites per window set to 0.1), CHIMAERA (scan triplets, fraction of variable sites per window set to 0.1) and Siscan (window of 200 bp, step size of 20 bp, use 1/2/3 variable positions, nearest outlier for the 4^th^ sequence, 1000 *P* value permutations, 100 scan permutations).

### Other statistics

All qualitative variables with the exception of the sIA were compared using a Chi-squared test or the Fisher's exact test where appropriate; a *P* value ≤0.05 was considered to reflect significance. All computations were performed using R project software (http://www.r-project.org).

### Phylotaxonomics

**Figure 1:**
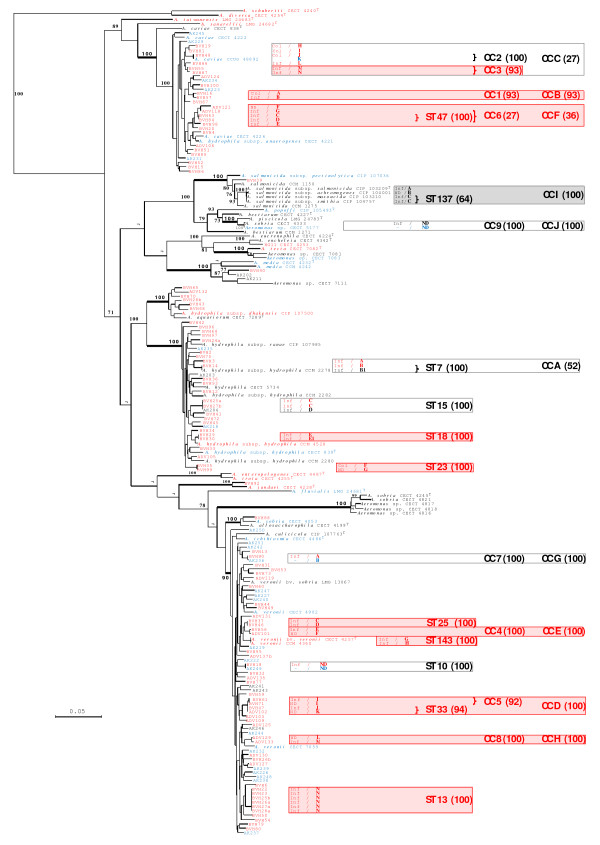
**Unrooted maximum-likelihood tree based on concatenated sequences of the seven housekeeping gene fragments (3993 nt).** The tree shows the structure of the studied *Aeromonas* spp. population, and the relative placement of human (red font), non-human animal (black font) and environmental (blue font) strains was indicated. The horizontal lines represent genetic distance, with the scale bar indicating the number of substitutions per nucleotide position. The numbers at the nodes are support values estimated with 100 bootstrap replicates. Only bootstrap values > 70 are indicated on the tree. The roots of the clades defined in 1 are represented by bold lines. MLPA clusters of strains sharing identical STs or grouped into CCs sharing at least 4 identical alleles at the 7 loci are indicated by frames (red frames for clusters of human strains, grey frames for clusters of non-human animal strains and uncolored frames for clusters of strains of various origins). In these frames, the following characteristics are indicated from left to right: (i) the strain’s clinical involvement when applicable as Inf for infection and Col for colonization; (ii) the *Swa*I pulsotype of the strains, with strains of identical pulsotypes designated by the same letter, strains with pulsotypes sharing more than 85% of their DNA fragments by A, A1, A2, … and strains with pulsotypes sharing no more than 70% of their DNA fragments by distinct letters, i.e., A, B, C, …; (iii) the names of STs shared by several strains; (iv) the names of CCs sharing at least 5 identical alleles at the 7 loci; and (v) the names of CCs sharing at least 4 identical alleles at the 7 loci. These ST and CC names are indicated to the right of the brackets grouping the strains with identical STs or belonging to the same CC and are followed by the bootstrap value (indicated in parentheses) supporting the corresponding MLPA cluster. (*) indicates that the relative position of the corresponding branch varied according to the method used. ND, not determined.

The population structure was inferred from multilocus phylogenetic analysis (MLPA) following reconstruction of the distance and ML trees from the concatenated sequences (alignment length of 3993 nt). The ML concatenated tree is shown in Figure 1, and the differences in the relative branching order between methods are also highlighted in Figure 1. The *Aeromonas* population was organized into 11 clades, which included 2 to 71 strains, with three major clades being observed (bootstrap values ≥ 90). The largest clade was comprised of 71 isolates, including 46 human, 5 animal and 20 environmental isolates, among which 4 were reference strains and three were type strains: *A. culicicola* CIP 107763^T^, *A. ichthiosmia* CECT 4486^T^, *A. veronii* biovar *sobria* LMG 13067 and *A. veronii* biovar *veronii* CECT 4257^T^; this was designated the *A. veronii* clade (Figure 1, Table 1). The two other major clades included 35 and 34 strains, respectively. They were referred to as the *A. hydrophila* clade (including strains *A. hydrophila* subsp. *hydrophila* CECT 839^T^, *A. hydrophila* subsp. *ranae* CIP 107985 and 33 other isolates) and the *A. caviae* clade (including *A. caviae* CECT 838^T^, *A. hydrophila* subsp. *anaerogenes* CECT 4221 and 32 other isolates), respectively. Each of these clades contained strains from various sources, i.e., 25 human, 7 animal and 3 environmental strains in the *A. hydrophila* cluster and 24 human, 9 environmental and 1 animal isolate in the *A. caviae* cluster (Figure 1, Table 1). The remaining strains were distributed among eight minor clades (bootstrap values ≥ 90), and are presented in Table 1 and Figure 1. The relative branching order among clades remains uncertain for most nodes (Figure 1). The clades displayed a mean sequence divergence of 2.5%, but the *A. media* clade displayed higher genetic polymorphism than the other clades (5.8%). None of the isolates included in this study grouped with the type strains *A. bestiarum*, *A. diversa*, A*. encheleia*, *A. enteropelogenes*, *A. eucrenophila*, *A. fluvialis*, *A. popoffi*, *A. sanarellii*, *A. schubertii*, *A. taiwanensis*, and *A. trota*, or with the representative strain of hybridization group 11. Finally, strain CCM 1271 formed an independent phylogenetic branch that was clearly differentiated from related known species, particularly from *A. bestiarum*, the species name under which the strain is referenced in the Czech Collection of Microorganisms (Figure 1). A phylogenetic tree reconstructed for all the strains included in this study using a concatenated sequence of the 5 genes obtained for all of the strains also showed strain CCM 1271 to be unrelated to *A. bivalvium* CECT 7113^T^*, A. molluscorum* CIP 108876^T^*, A. simiae* CIP 107798^T^ and *A. rivuli* DSM 22539^T^ (see Additional file 1: Figure S1).

The multilocus sequence-based phylogeny supported the current taxonomy of the genus. In addition, both the high level of concatenated sequence divergence observed in the *A. media* cluster and the comparison of the subtree topology for clusters including closely related known species, such as *A. eucrenophila*-*A. encheleia*-*A. tecta*, suggested that the *A. media* clade may constitute a polyphyletic cluster containing taxa that have yet to be described. Strain CCM 1271, showing a clearly segregated phylogenetic position in the MLPA, also likely represents an unknown *Aeromonas* taxon.

### Genetic diversity

The number of different alleles for the 7 loci varied from 111 (*rpoB*) to 160 (*dnaK*) (Table 3). This significant variation (*P* value = 10^-9^) suggested distinct mutation rates among the loci. The equivalent mol% G + C content ranged from 55.8 (*tsf*) to 62.6% (*radA*) for all loci with the exception of *zipA*, which exhibited a lower mol% G + C content of 52.4%. The mean genetic diversity among strains was high for the whole genus, and of the 3 main clades, *A. caviae* displayed the lowest genetic diversity (h) for all genes (Table 3). The rate of polymorphic sites varied significantly between the *A. caviae, A. hydrophila* and *A. veronii* clades for all loci except for *rpoB*, with *A. caviae* being the clade that showed the lowest number of polymorphic sites for all loci (Table 3). The rates of non-synonymous versus synonymous substitutions (dN/dS ratio) were rather low, except for *zipA*, indicating that among the 7 loci, only one locus had been subjected to strong positive selective pressure (Table 3).

**Table 3:** Sequence analysis for the seven loci of the MLSA scheme

**Locus**	**Genus or Clade**	**No. of alleles (% per clade)**	**No. (%) of polymorphic sites**	**Genetic diversity (h)^a^**	**No. of non-synonymous codons**	**dN/dS ratio**	**G+C%^b^**
*dnaK*	Genus (n=191)	160	273 (33.5)	0.9970	56	0.27	58.8
	*A. caviae* (n=34)	26 (76.5)	70 (8.6)	0.9697	3	0.006	60.1
	*A. hydrophila* (n=35)	29 (82.9)	87 (10.7)	0.9882	0	0	59.5
	*A. veronii* (n=71)	57 (81.4)	119 (14.6)	0.9901	0	0	57.8
	*P* value	NS	6.10^-8^	-	0.036	-	-
*gltA*	Genus (n=191)	141	160 (30.5)	0.9900	21	0.048	61.5
	*A. caviae* (n=34)	18 (52.9)	28 (6.1)	0.8324	4	0.089	61.8
	*A. hydrophila* (n=35)	26 (74.3)	41 (8.9)	0.9714	1	0.006	62.5
	*A. veronii* (n=71)	50 (75.7)	70 (15.1)	0.9735	1	0.002	60.8
	*P* value	NS	6.10^-7^	-	NS	-	-
*gyrB*	Genus (n=191)	154	278 (35.1)	0.9966	39	0.035	59.1
	*A. caviae* (n=34)	26 (76.5)	58 (7.3)	0.9786	2	0.004	60.7
	*A. hydrophila* (n=35)	28 (80.0)	92 (11.6)	0.9885	3	0.005	59.6
	*A. veronii* (n=71)	55 (82.9)	137 (17.3)	0.9884	7	0.012	58
	*P* value	NS	10^-10^	-	NS	-	-
*radA*	Genus (n=191)	148	194 (46.6)	0.9955	30	0.061	62.6
	*A. caviae* (n=34)	23 (67.6)	28 (6.7)	0.9661	1	0.007	63.4
	*A. hydrophila* (n=35)	28 (80.0)	61 (14.5)	0.9832	5	0.029	64.6
	*A. veronii* (n=71)	50 (71.4)	66 (15.7)	0.9801	6	0.009	61.1
	*P* value	NS	10^-14^	-	NS	-	-
*rpoB*	Genus (n=191)	111	98 (23.0)	0.9846	6	0.004	57
	*A. caviae* (n=34)	13 (38.2)	18 (4.2)	0.7683	1	0.013	58.7
	*A. hydrophila* (n=35)	24 (68.6)	24 (5.6)	0.9681	0	0	56.3
	*A. veronii* (n=71)	31 (44.3)	25 (5.9)	0.9528	0	0	56.4
	*P* value	0.02	0.31	-	NS	-	-
*tsf*	Genus (n=191)	118	177 (27.1)	0.9844	30	0.068	55.8
	*A. caviae* (n=34)	16 (47.1)	16 (2.3)	0.9073	1	0.015	56.5
	*A. hydrophila* (n=35)	21 (60.0)	24 (3.4)	0.9445	1	0.008	55.9
	*A. veronii* (n=71)	37 (52.9)	79 (11.8)	0.9288	9	0.032	55.3
	*P* value	NS	3.10^-5^	-	0.004	-	-
*zipA*	Genus (n=191)	137	380 (70.8)	0.9929	130	0.333	52.4
	*A. caviae* (n=34)	20 (58.8)	98 (18.3)	0.9358	31	0.276	52.9
	*A. hydrophila* (n=35)	25 (71.4)	31 (5.8)	0.9697	6	0.071	53.6
	*A. veronii* (n=71)	46 (66.2)	50 (9.3)	0.9718	12	0.158	51.3
	*P* value	NS	3.10^-5^	-	10^-5^	-	-

^a^Mean genetic diversity (H) among strains for the whole genus: 0.9916 ± 0.0020 and for the main 3 *A. hydrophila*, *A. caviae*, *A. veronii* clades: 0.9724 ± 0.0055, 0.9083 ± 0.0301 and 0.9694± 0.0082, respectively.

^b^Mean G+C% values for the 7 loci: 58.15% for the 191 *Aeromonas* spp. strains of this study, 59.2% for *A. caviae* clade, 58.9% *A. hydrophila* clade and 57.2% *A. veronii* clade.

NS: not significant, dN/dS: rate of non-synonymous versus synonymous substitutions.

-: not determined.

*P* value indicates result of statistical tests for data comparison between clades.

### Multilocus sequence typing, genomic relationships and origin of the strains

The multilocus sequence dataset for the 191 strains contained 175 sequence types (STs), 164 (93.7%) of which were identified only once. ST diversity was 0.92 per strain for the genus *Aeromonas*, confirming its exceptionally high level of population diversity, which was also observed in the *A. caviae*, *A. hydrophila* and *A. veronii* clades, which exhibited 0.97, 0.86 and 0.87 ST per strain, respectively. The largest ST group included 6 strains of the *A. veronii* clade. A total of 10 other STs were shared by a maximum of 3 strains (Table 1, Figure 1).

The clustering of STs in CCs sharing at least 5 identical alleles at the 7 loci revealed 9 CCs, which grouped a maximum of 3 strains. These CCs corresponded to MLPA clades supported by high bootstrap values ≥ 92%, except for CC “6” (Figure 1, Table 1). Using a less stringent definition of CCs (4 identical allele at the 7 loci) did not significantly change the population clustering, confirming that the high genetic diversity of the population was equally expressed at each locus (Table 1, Figure 1 and 2).

Links among strains sharing the same ST and strains grouped into CCs were further investigated by comparing their geographic origins and isolation dates and using PFGE. The genomic macro-restriction digest with the endonuclease *Swa*I produced PFGE patterns that comprised of an average of 18 bands suitable for strain comparison (data not shown). The strains grouped within each of these clusters showed distinct pulsotypes and/or were of distinct geographic origin and, in some cases, had been isolated over a long time period. For example, ST7 included strains BVH14 and CCM 2278, sharing more than 85% of their DNA fragments in the PFGE analysis, which were isolated in France in 2006 and in California in 1963, respectively (Table 1, Figure 1). Of particular note, the largest ST found in this study, ST13, included 6 strains with identical pulsotypes, despite being isolated in 2006 from distant sites (e.g., La Réunion Island in the Indian ocean and 2 distant regions in mainland France). Finally, we observed that the type strains of *A. salmonicida* subsp. *masoucida* and *A. salmonicida* subsp. *smithia* purchased from the Collection of the Institut Pasteur showed identical STs and pulsotypes; this questionable result should be considered with caution until a further control analysis is performed in strains ordered from another collection.

Comparison of the overall diversity observed according to the origin of the strains within the 3 main clades showed that the number of STs per strain differed significantly between the groups of clinical and environmental isolates (0.875 and 1, respectively; *P* value = 0.036). This difference also reached the level of significance among the *A. veronii* group (*P* value = 0.049). A few robust clusters of strains were shown to group isolates from the same host origin, which primarily grouped strains of human origin (Figure 1, Table 1). Out of the 43 geographically and/or genomically unrelated strains belonging to the 3 main clades included in either identical ST groups or in clonal complexes, only 3 originated from the environment, and 2 were involved in animal diseases (Table 1 and Figure 1). This difference in the distribution of environmental/animal and human clinical strains was statistically significant (*P* value = 5.10^-4^) for the 3 main clades and for the *A. veronii* (*P* value = 0.02) and *A. caviae* (*P* value = 0.05) clades.

Finally, a non-random distribution of strains was observed among the different CCs according to their site of isolation and/or colonizing/pathogenic status. CC “C” grouped 3 out of the 5 non-pathogenic, colonizing *A. caviae* strains in the dataset, and this rate was significantly different from that of the non-pathogenic *A. caviae* strains found outside of the CC (*P* value = 0.04) (Table 1, Figure 1). In contrast, some other clusters included strains involved only in infectious processes (Table 1, Figure 1). Finally, the *A. veronii* ST13 cluster appeared to be associated with a particular type of disease, i.e., wound infection. Indeed, 5 out of the 12 *A. veronii* strains in the dataset involved in wound infection were grouped into this cluster, representing a frequency that was significantly different from the rest of the *A. veronii* population (*P* value = 0.0001).

### Recombination events in the aeromonad population

The sIA value was 0.30 at the genus level, ranged from 0.15 to 0.42 at the clade level and was significantly different from 0, indicating the existence of significant linkage disequilibrium, showing that the studied *Aeromonas* population was not panmictic but clonal. Events of recombination among the clonal population were then analyzed via RDP, decomposition analysis and phylogenetic incongruence.

Considering the recombination events detected using at least 4 methods of the RDP software, 14 types of recombination events leading to 166 recombinant sequences were detected among the population and are detailed in an additional table (Additional file 2: Table S2). All but two loci (*radA* and *rpoB*) were affected by recombination events that occurred in 89 STs (50.9%). *dnaK* and *gyrB* were the most affected loci (4 events each, 75 and 13 recombinant sequences, respectively), followed by *tsf* and *zipA* (3 events each, 73 and 5 recombinant sequences, respectively) and *gltA* (1 event and 3 recombinant sequences) (data not shown). One to four types of significant recombination events occurred in most clades, except for the *A. hydrophila*, *A. piscicola* and *A. tecta* clades and the *A. fluvialis* type strain and strain CCM 1271. Five events could not be significantly linked to parental sequences, suggesting the occurrence of transfer from strains that are not represented in our collection.

Recombination was also investigated for the 3 main clades via split decomposition in the concatenated sequences (Additional file 3: Figure S3 a-c). Most of the STs were not affected by recombination, and the trees showed a limited parallelogram formation, notably including *A. hydrophila* STs (Additional file 3: Figure S3 b). Split decomposition detected more recombination events within the CCs, particularly for STs in the *A. caviae* clade (Additional file 3: Figure S3 a).

Distance and ML trees were reconstructed for each of the 7 genes and compared to the concatenated sequence-based trees. For all genes and phylogenetic methods, single locus phylogenies (SLPA) displayed lower bootstrap values than MLPA trees (data not shown). Moreover, differences in branching order were observed in SLPA, suggesting the occurrence of recombination events (data not shown). In detail, phylogenetic discordance was observed for 11 strains based on single-gene phylogenetic analysis: all of these strains grouped in a robust cluster that was different from the cluster defined based on the 6 other genes or the concatenated sequence (shown in bold text in Table 1). Identical alleles were observed in strains belonging to different MLPA clusters, i.e., *gyrB* allele 83, common to the two environmental strains *A. veronii* strain AK250 and *A. hydrophila* strain AK218; *zipA* allele 97, common to the *A. media* and *A. enteropelogenes* type strains; and *zipA* allele 94, which was identical in the *A. caviae* type strain and *A. salmonicida* strain CIP104001 (Table 1). In addition, strain BVH53 belonged to the *A. veronii* clade in the MLPA, while it was robustly grouped with the *A. jandaei* type strain in the *gyrB*-based phylogeny (bootstrap value of 100% in both the ML and distance-based trees) (data not shown). Similarly, among the isolated strains, the *A. fluvialis* type strain showed a divergent phylogenetic position between the *gltA*-based tree, where it robustly grouped with the *A. schubertii* type strain, and other gene-based phylogenies or the MLPA. Finally, strain BVH39 grouped within the *A. salmonicida* clade in the multilocus tree, while it was excluded from the corresponding clade defined in the *dnaK*-based tree. These phylogenetic incongruities revealed a total of 12 recombination events (0.9% of the sequences), which occurred in 11 strains (4, 3 and 4 strains of human, animal and environmental origin, respectively) (5.8% of the total strains) and concerned 5 out of the 7 genes addressed in our MLSA scheme, i.e., *dnaK* (1 strain), *gltA* (1 strain)*, gyrB* (4 strains), *tsf* (3 strains), and *zipA* (3 strains) (Table 1). Multilocus phylogenetic trees reconstructed excluding the strains subjected to recombination showed increased bootstrap values for the *A. veronii* clade (90 to 100%) as well as for most interclade nodes, confirming that recombination distorted the MLPA (data not shown). Despite its relatively low frequency of occurrence in the genus *Aeromonas*, recombination may account, at least in part, for some controversial taxonomic issues. For example, strain CCM 1271 is closely related to *A. bestiarum* in the *gyrB*-based phylogenetic tree (data not shown), whereas it is clearly individualized from this species in the MLPA.

## Discussion

In this study, we investigated the genetic diversity and population structure linked with strain origin using MLSA. This work, based on a large collection of clinical isolates, i) estimated high genetic diversity, recombination rates and horizontal gene transfer in *Aeromonas* species, which together highlighted the mode of evolution in this group; ii) showed small clusters of strains associated with human infection; and iii) provided phylotaxonomic data that helped clarify the confusing taxonomy of the genus *Aeromonas* in relation to other works [15,16,28]. These results are further discussed below.

### A MLSA scheme for studying *Aeromonas* spp. population structure

This was the 3^rd^ multilocus scheme proposed for studying *Aeromonas* spp. in 2011 [15,16]. These three studies analyzed different populations of aeromonads with different set of genes and different objectives. The 1^st^ MLSA scheme was developed for analyzing *Aeromonas* phylogeny and attempting to resolve the taxonomic controversies within this genus [16]. The 2^nd^ was developed to achieve precise strain genotyping and phylogenetic analysis of outbreak traceability and genetic diversity and was based on strains isolated from fish, crustaceans and mollusks [15]. The MLSA that we have presented here improved the understanding of human aeromonosis by addressing a large population that included both clinical and environmental strains from diverse geographic sources. The overall collection represented different lifestyles encountered in the genus: free living or associated with humans or cold-blooded animals. The clinical strain collection was representative of the French epidemiology because it resulted from a systematic prospective nationwide record and was associated with well-documented clinical reports [17]. The size of the collection was increased by including strains from various collections, most of which came from animal and environmental sources, so that the overall collection studied herein totaled 195 strains, which is a greater number compared to the two other MLSA studies on *Aeromonas*[15,16]. Our MLSA scheme was suitable for analysis of the whole genus *Aeromonas*, with the exception of four species: *A. bivalviumA. molluscorumA. simiae* and *A. rivuli*, for which only 6 genes could be analyzed. This MLPA allowed structuring the population into 3 main clades, designated *A. veroniiA. hydrophila* and *A. caviae*, because they contained the type strains of these species. Despite the fact that the number of isolates in the main clades was high compared to the study by Martino et al. [15] and similar to other studies [e.g., [29], the number of strains in some clades remained rather limited (e.g., *A. caviae*: 34 strains), and our results should be confirmed in a larger population. For this purpose, the population results and MLSA scheme have been deposited in a public database (PubMLST: http://pubmlst.org/software/database/bigdb/) [30]. Nevertheless, our results provided interesting insight into the genetic diversity and structure of the *Aeromonas* population encountered in clinical infections as well as the mode of evolution of this population.

The MLSA scheme presented here included 7 housekeeping genes, which were sporadically distributed among the genome and, thus, can reasonably be assumed to not be associated with mobile genetic elements. The 7 genes encoded components of different metabolic pathways and were characterized by different mutation rates and low positive selective pressure, indicating predominantly neutral evolution of the loci. In our MLSA scheme, we propose the use of 4 genes not previously included in the 2 other MLSA schemes (*dnaKradAtsf* and *zipA*) for inferring the *Aeromonas* population structure. Despite its markedly lower mol% G + C content compared to other loci and to the mean value for the genus *Aeromonas* inferred from the *A. hydrophila* ATCC 7966^T^*A. salmonicida* A449*,* and *A. caviae* Ae398 genomes (approximately 61.5%) and the *A. veronii* B565 genome (58.8%) [2,31-33], *zipA* was included in the MLSA scheme because the *zipA*-based phylogenetic tree was mostly congruent with those obtained for other genes (data not shown). This suggested that this gene likely originated from a distant genus through lateral genetic transfer, though it was likely acquired by a common ancestor of the population studied. Altogether, the 3 available multilocus schemes for *Aeromonas* indicated 16 distinct genes, i.e., *atpD, dnaJ, dnaK, dnaX, gltA, groL, gyrA, gyrB, metG, ppsA, radA, recA, rpoB, rpoD, tsf,* and *zipA,* that will offer the possibility of performing accurate analyses in *Aeromonas*.

### Mode of evolution

Both the genetic and ST diversity per strain were observed to be exceptionally high in the genus *Aeromonas* and were much higher than observed for many other environmental bacteria [9,11,34]. Although strains from countries other than France only represented approximately 25% of the total strains of our dataset, the high level of genetic diversity observed validated the non-redundancy and representativeness of our population. Given that some geographically distant strains were very closely related (e.g., BVH 14 and CCM 2278, (Figure 1 and Table 1)), the global genetic diversity of this group may be reflected in that of a rather small sampling population, as observed in other reports on water-living species with high genetic diversities, such as *Pseudomonas aeruginosa*[35]. However, further analysis will be required to confirm this hypothesis. High diversity was observed in the 3 main *A. caviae, A. hydrophila* and *A. veronii* clades; however, the genetic characteristics and population structure of the *A. caviae* clade were outstanding. Compared to the other two mains groups, the *A. caviae* clade showed a lower genetic diversity, indicated both by its genetic diversity (h) and lower number of polymorphic sites. However, *A. caviae* exhibited the highest dN/dS ratio for 4 genes. These results suggested that *A. caviae* strains have experienced less genetic variation, but when such variations have occurred, the mutations have more often been non-synonymous. Positive selective pressure or genetic hitchhiking is unlikely to explain this phenomenon. In this context, the occurrence of deleterious mutations linked to demographic effects experienced by the population represents a hypothesis that can explain the genetic particularities of *A. caviae*. The high genetic diversity in the genus, as observed by other researchers as well [15,36] reflects the behavior of aeromonads as water-living bacteria. In fact, *Aeromonas* represent an outstanding example of generalist bacteria displaying genetic and genomic traits associated with this lifestyle and their ability to adapt to diverse niches, i.e., a relatively large genome (4.7 Mb) [2], high genetic diversity, significant rate of horizontal gene transfer of housekeeping genes (5.8% in our population), a significant number of ribosomal operons that are sometimes heterogeneous and submitted to cross-over events [37-39], genomic and phenotypic plasticity [2] and a great ability to adapt to new niches. All of this diversity corresponded to structuring in terms of complexes of species rather than species *sensu stricto*[40]. The wide range of genetic repertoires included in these complexes of species may constitute a potential reservoir for the emergence of future specialists via a speciation process related to selective pressure within a narrow niche. The complex and confusing systematics of the genus *Aeromonas* may result, at least in part, from the structure in species complexes in which speciation is progressing locally. For example, the species status of *A. allosaccharophila*, a clade closely related to *A. veronii*, has long been controversial, and evidence indicating whether this represents a definitive species has varied according to the methods used and the housekeeping genes analyzed [16,28,41-45]. If speciation is currently in progress for *A. allosaccharophila*, it could explain these controversial data, as highlighted by Silver [28] or as observed in other genera (e.g., the *Burkholderia cepacia* complex [46]). In contrast, *A. salmonicida* could represent an example of a fish-adapted species subjected to some costs of specialization (e.g., being non-motile, having the ability to growth at 25°C but not at 37°C) [33]. In this study, *A. caviae* appeared to have exceptional genetics compared to *A. veronii* or *A. hydrophila*. The hypothesis of a population bottleneck related to adaptation to a specialized niche, such as the gut, which is a more frequent niche for *A. caviae* compared to other aeromonads, should be emphasized. In fact, compared to *A. veronii* and *A. hydrophila, A. caviae* is preferentially found in the gut, as highlighted by the higher frequencies of gastroenteritis and bacteremia infections originating from the gut [17] and the higher density of *A. caviae* in wastewater inflows than outflows [47,48].

The diversity was significantly lower among the clinical strains than the environmental strains, which is compatible with the hypothesis that some clinical strains may correspond to niche-adapted subpopulations. Robust MLPA clusters of strains with identical STs or belonging to CCs were identified among the population, mainly among the 3 main clades this study. Each of these clusters included a limited number of strains (2 to 6 strains) that were further shown to be unrelated based on epidemiological data and/or PFGE results, and 52 out of the 191 fully analyzed strains (27.2%) were involved in these clusters. Twelve clusters grouped strains from a unique host, i.e., a fish-associated subset within *A. salmonicida* and 11 human-associated subsets within the *A. veronii* (n = 6), *A. caviae* (n = 3) and *A. hydrophila* (n = 2) clades. Nine of these subsets included only disease-associated strains. Notably, all of the *A. veronii* human-associated clusters were disease associated. Among these clusters, ST13, which was shared by 6 strains of human origin and was mainly recovered during wound infections, may reflect a host (niche)-adapted pathogenic cluster among the *A. veronii* clade, which was otherwise characterized by high genetic diversity. The striking, unique PFGE pattern and ST may reflect the adaptation of this cluster to a niche in which genetic and genomic variability is not permitted due to strong constraints. However, because of the small number of strains included in these clusters, an increased number of strains should be studied to confirm whether specific lineages or CCs are more likely to contain clinical isolates or be associated with a specific illness.

The present study showed a relatively low frequency of recombination events compared to previous studies [15,28]. This result may originate in the differences between these studies in the genes evaluated and the population sampling strategies employed. The population sample studied by Martino et al. differed significantly from ours, as most of their isolates were obtained from fish, crustaceans or mollusks [15]. Silver et al. deliberately included a very small number of isolates (n = 12) of host-associated strains (e.g., only strains isolated from leeches, human wounds or human feces), which may constitute a recruitment bias because these strains may be host adapted [28]. Interestingly, the recombination events encountered in our study were predominantly observed within clonal complexes (e.g., CC “D”, grouping *A. veronii* strains recovered during human diarrhea episodes), which supported the previous hypothesis of the study by Silver et al. [28].

### Taxonomic considerations

MLPA may be helpful for identification purposes. Indeed, strains that have previously rarely been reported in the literature were recognized among the study population, among which an *A. jandaei* isolate from a human urinary tract infection and an *A. allosaccharophila* strain recovered during human bacteremia were particularly remarkable. Moreover, MLPA may allow the correct identification of strains deposited in strain collections under erroneous or incomplete nomenclature, as observed for *A. sobria* CECT 4333 and *Aeromonas* sp. CECT 5177, which most likely belong to the *A. piscicola* species.

Multilocus sequence-based phylogeny supported recent taxonomic changes in the genus *Aeromonas*. First, several recently characterized species were clearly individualized in the 7 gene-based phylogenetic trees, such as *A. taiwanensisA. sanarellii* and *A. fluvialis*[49,50]. The proposal of *A. diversa*, including *Aeromonas* sp. HG13, referred to as *Aeromonas* group 501, as a distinct species from *A. schubertii* was supported in the MLPA by the clearly individualized phylogenetic positions observed for these two species [51]. Moreover, several taxonomic reappraisals were confirmed by our approach, as observed and discussed in the MLPA study by Martinez-Murcia et al. [16,52]. In addition, evidence previously suggesting that *A. hydrophila* subsp. *anaerogenes* and *A. caviae* are conspecific was confirmed here by the *A. hydrophila* subsp. *anaerogenes* strain CECT 4221 that was found to belong to the *A. caviae* clade [53]. All of these observations showed that the MLSA scheme appeared to be a strongly informative tool that should be included within the methods used for polyphasic taxonomic analysis in the genus *Aeromonas*. Thus, this MLSA scheme may provide additional arguments regarding controversial issues recently reviewed by Janda & Abbott [1]. *A. ichthiosmia*, which is considered to be a later synonym of *A. veronii*[42], clearly grouped in the *A. veronii* clade. *A. encheleia* showed a low level of genetic divergence at the 7 loci and grouped in a tight and robust clade with HG11, providing additional arguments for their unification. *A. allosaccharophila*, whose existence is still controversial, occupies a robust position that is closely related, but external to the *A. veronii* clade, in favor of the separation of the two taxa. However, the taxonomic level of the new taxon, if proposed, still has to be defined due to conflicting DNA-DNA hybridization values compared to the *A. veronii* type strain according to the study considered [42,54]. Finally, the *A. caviae* type strain occupies a position external to those of other members of the *A. caviae* clade in the MLPA-based tree. This observation warrants further investigation due to the taxonomic value of the MLSA scheme demonstrated here. Of note, only 2 genes (*gyrB* and *rpoB*) from *A. sharmana*, a species that was shown not to belong to the genus *Aeromonas* and is awaiting reassignment, could be amplified using the primers employed in this study [55,56].

## Conclusions

Evolution in the genus *Aeromonas* has involved the combined effects of mutations and recombination events, resulting in an exceptionally high genetic diversity. We propose a hypothetical mode of evolution in aeromonads based on global organization into a complex of species, with local emergence of more specialized clones. This specialization in process is suggested by the co-existence of i) specialized species *sensu stricto*, such as *A. salmonicida*, ii) clades showing genetic traits associated with speciation and adaptation to a specific niche, such as *A. caviae*, and iii) diverse subsets of strains that may be host adapted and/or “disease specialized”. The MLSA scheme developed herein in a large and diverse population of strains helped shed light on the unclear relationships among *Aeromonas* strains and aeromonosis. However, certain clades and the host- and/or disease-associated subsets of strains detected in this study included a limited number of strains. As a consequence, additional studies are required to increase the size of the analyzed population and to confirm these results. Further work including a virulence analysis focusing on human clinical clusters is also needed. Finally, the MLSA scheme proposed here appeared to be useful for taxonomic studies in the genus *Aeromonas*.

## Authors’ contributions

Conceived and designed the study: EJB, HM, BL. Designed and performed the acquisition of clinical data and isolate collection: colBVH, AK, BL. Performed the microbial and molecular genetic analyses: FR (primer design, MLSA and MLPA, PFGE), AK (curator of the clinical isolates collection, *rpoB* analysis). Analyzed and interpreted the data: FR, BL (all data), HM (PFGE and MLPA), EJB (MLSA), BL (statistics). Drafted the paper: HM, BL. Helped to draft the manuscript: FR. Critically revised the manuscript: EJB. All authors read and approved the final manuscript.

## Supplementary Material

Additional file 1 **Figure S1.** Unrooted maximum-likelihood tree based on concatenated sequences of five housekeeping gene fragments (*gltA, gyrB, rpoB, tsf, zipA, *2724 nt). The horizontal lines indicate genetic distance, with the scale bar indicating the number of substitutions per nucleotide position. The numbers at the nodes are support values estimated with 100 bootstrap replicates. Only bootstrap values > 70 are shown on the tree. The clades defined in Table 1 are indicated with brackets at the top right of the figure. Only type strains and reference strains are represented in the tree. Click here for file

Additional file 2 **Table S2.** Recombination event types and recombinant sequences. Click here for file

Additional file 3 **Figure S3.** SplitsTree decomposition analyses of the MLSA data for strains belonging to the*A. caviae***(a)**, *A. hydrophila***(b)** and*A. veronii***(c)** clades. The distance matrix was obtained from the allelic profiles of the sequence types (ST). A network-like graph indicates recombination events. Star-like radiation from the central point indicates an absence of recombination. The names of eBURST clonal complexes (CCs), as defined in the text and in Table 1, are indicated near the corresponding STs. The number of strains sharing an identical ST is indicated below the ST number in brackets. Type strain STs are indicated by dots. Click here for file
