# Supplementary figures and images for: Multilocus genetics to reconstruct aeromonad evolution
Source: BMC Microbiol. 2012 Apr 30;12:62. doi: 10.1186/1471-2180-12-62 (PMC3487998; doi:10.1186/1471-2180-12-62)

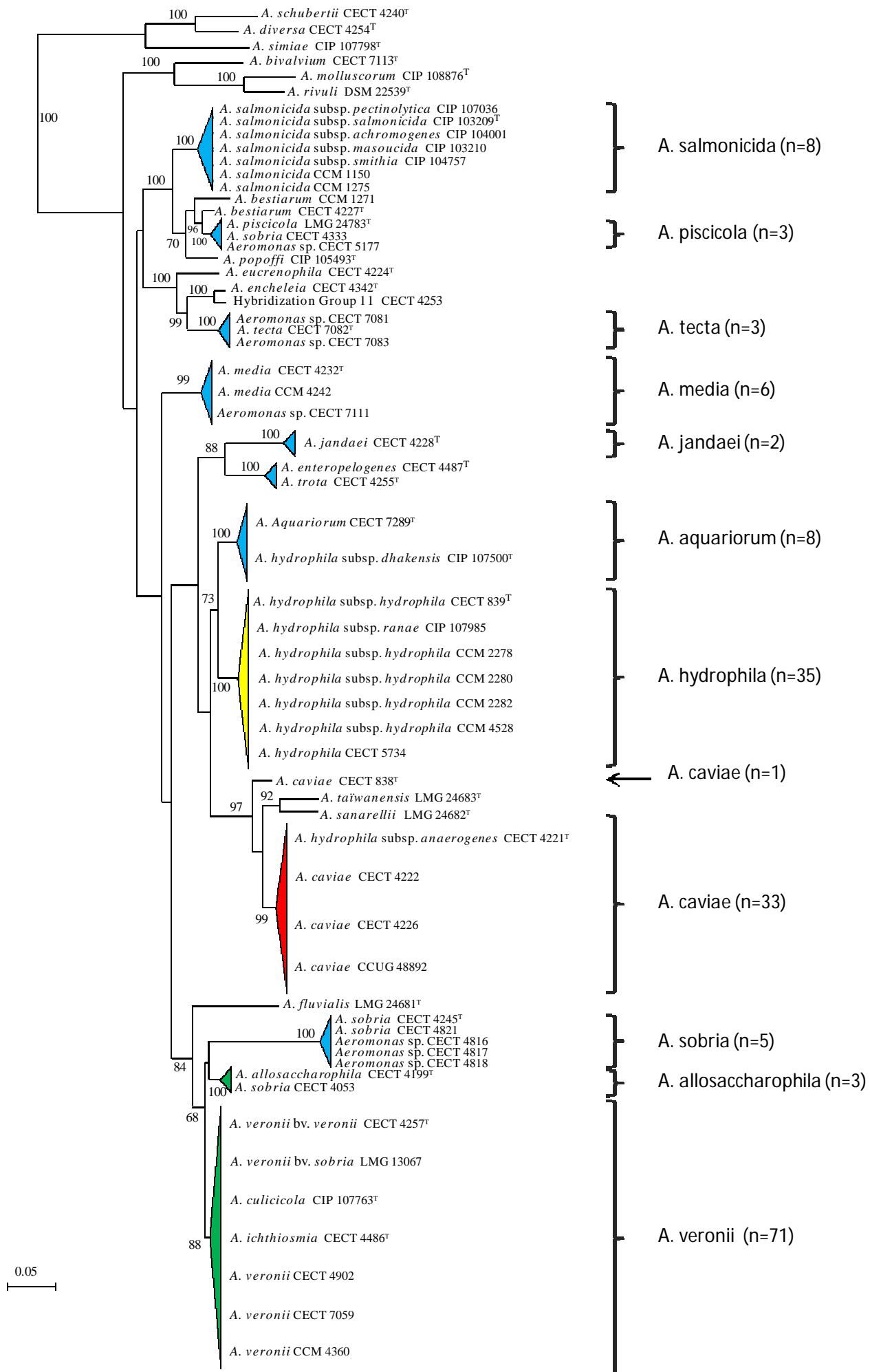

Supplementary Fig. 1

Supplement: Additional file 1 — Figure S1. Unrooted maximum-likelihood tree based on concatenated sequences of five housekeeping gene fragments (gltA, gyrB, rpoB, tsf, zipA, 2724 nt). The horizontal lines indicate genetic distance, with the scale bar indicating the number of substitutions per nucleotide position. The numbers at the nodes are support values estimated with 100 bootstrap replicates. Only bootstrap values > 70 are shown on the tree. The clades defined in Table 1 are indicated with brackets at the top right of the figure. Only type strains and reference strains are represented in the tree. [file 1471-2180-12-62-S1.pdf]

*A. caviae*  
CECT 838<sup>T</sup>

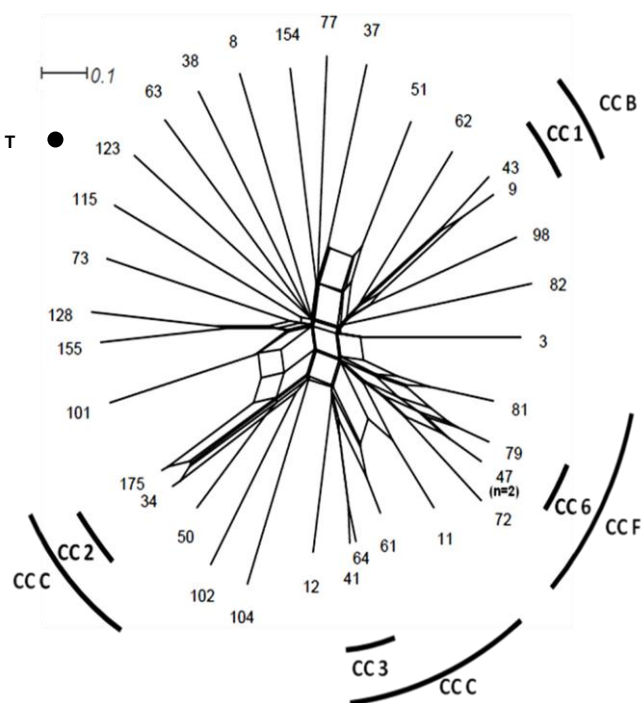

(a)

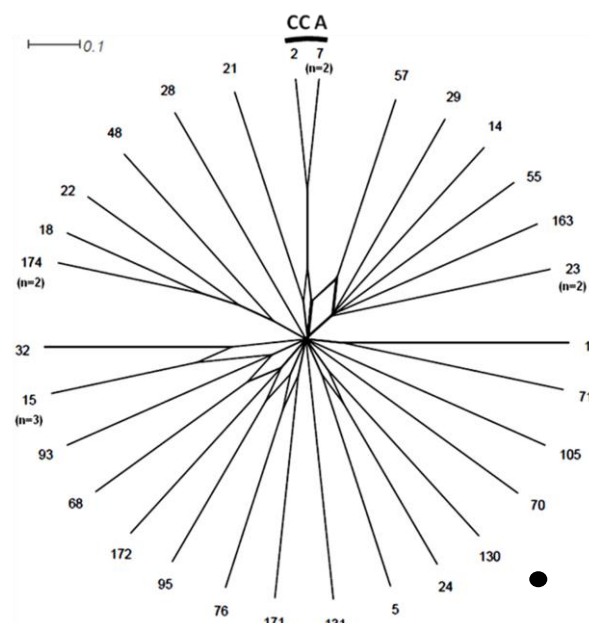

(b)

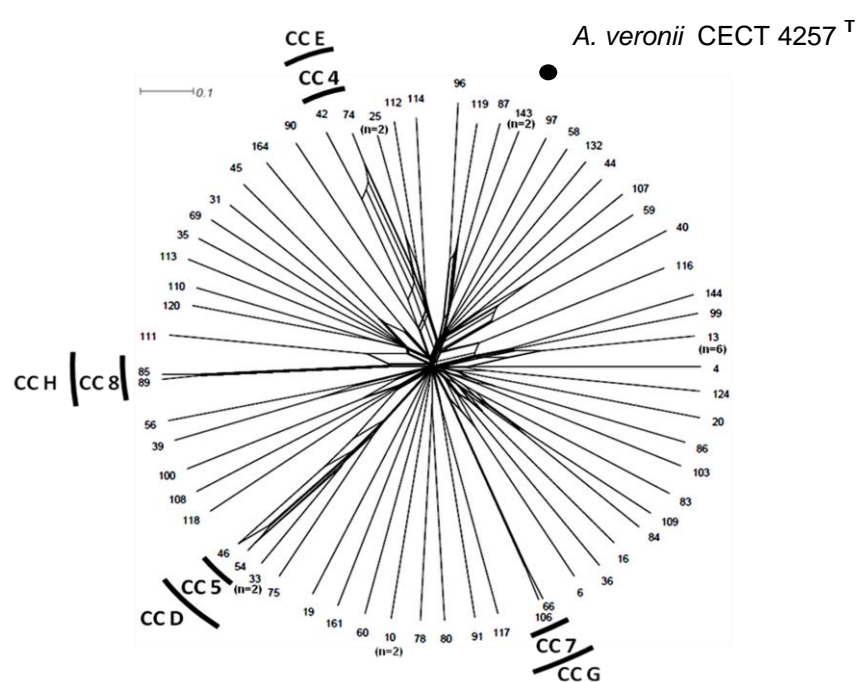

(c)

Supplement: Additional file 3 — Figure S3. SplitsTree decomposition analyses of the MLSA data for strains belonging to theA. caviae(a), A. hydrophila(b) andA. veronii(c) clades. The distance matrix was obtained from the allelic profiles of the sequence types (ST). A network-like graph indicates recombination events. Star-like radiation from the central point indicates an absence of recombination. The names of eBURST clonal complexes (CCs), as defined in the text and in Table 1, are indicated near the corresponding STs. The number of strains sharing an identical ST is indicated below the ST number in brackets. Type strain STs are indicated by dots. [file 1471-2180-12-62-S3.pdf]
