# Supplementary material for: Multilocus genetics to reconstruct aeromonad evolution
Source: BMC Microbiol. 2012 Apr 30;12:62. doi: 10.1186/1471-2180-12-62 (PMC3487998; doi:10.1186/1471-2180-12-62)
Supplement: Additional file 2 — Table S2. Recombination event types and recombinant sequences. [file 1471-2180-12-62-S2.doc]

**Supplementary Table 1.** Recombination event types and recombinant sequences.

| Number of methods with statistically significant detection of  recombination events | Event type  (number of recombinant sequences) | Total of  recombinant sequences |
| --- | --- | --- |
| 7 | 1(5), 2(1), 3(1), 4(1), 5(3), 6(3), 8(1) | 15 |
| 6 | - | - |
| 5 | 16(1) | 1 |
| 4 | 7(1), 9(1), 11(68), 13(3), 20(8), 24(69) | 150 |
| 3 | 12(14), 14(1), 17(5), 22(17), 25(2), 29(2) | 41 |
| 2 | 10(1), 18(8), 19(2), 23(1), 27(3), 28(1), 30(3), 38(9) | 28 |
| 1 | 15(1), 21(2), 26(3), 31(1), 32(1), 33(2), 34(7), 35(7), 36(4), 37(6) | 34 |
